# Computerised tomography features of giant cell tumour of the knee are associated with local recurrence after extended curettage

**DOI:** 10.1007/s00264-021-05260-6

**Published:** 2021-11-16

**Authors:** Lenian Zhou, Hongyi Zhu, Shanyi Lin, Hanqiang Jin, Zhaoyuan Zhang, Yang Dong, Qingcheng Yang, Changqing Zhang, Ting Yuan

**Affiliations:** 1 Department of Orthopaedics, Shanghai Jiao Tong University Affiliated Sixth People’s Hospital, 600 Yishan Road, Shanghai, 200233 China; 2 Institute of Microsurgery On Extremities, Shanghai Jiaotong University Affiliated Sixth, People’s Hospital, Shanghai, 200233 China

**Keywords:** Giant cell tumour of bone, Cortical bone, Tomography, X-ray computed, Recurrence, Curettage

## Abstract

**Background:**

Extended curettage has increasingly become the preferred treatment for giant cell tumour of bone (GCTB), but the high recurrence rate after curettage poses a major challenge for orthopaedic surgeons. Computed tomography (CT) is valuable in the evaluation of GCTB. Our aim was to identify specific features of GCTB around the knee in pre-operative CT images that might have prognostic value for local recurrence.

**Methods:**

We retrospectively analyzed data from 124 patients with primary GCTB around the knee who underwent extended curettage from 2010 through 2019. We collected demographic, clinical, and therapeutic data along with several CT-derived tumour characteristics. CT-derived tumor characteristics included tumour size, the distance between the tumour edge and articular surface (DTA), and destruction of posterior cortical bone (DPC). Akaike information criterion (AIC) was used to select which variables to enter into multivariate logistic regression models and to determine significant factors affecting recurrence.

**Results:**

The total recurrence rate was 21.0% (26/124), and the average follow-up time was 69.5 ± 31.2 months (24–127 months). Age, DTA (< 2 mm), and DPC were significantly related to recurrence, as determined by multivariate logistic regression. The C-index of the final model was 0.79 (95% CI: 0.71 to 0.88), representing a good model for predicting recurrence.

**Conclusion:**

Identifying certain features of GCTB around the knee on CT has prognostic value for patients treated with extended curettage. A three-factor model predicts tumour recurrence well after extended curettage.

**Supplementary Information:**

The online version contains supplementary material available at 10.1007/s00264-021-05260-6.

## Introduction

Giant cell tumours of bone (GCTBs) are locally aggressive and intermediate (rarely metastasizing) bone tumours that usually occur in young individuals aged 20 to 45 years [1]. GCTBs account for approximately 20% of all musculoskeletal tumours in Asian patients [2]. GCTBs are typically found in the epi-metaphyseal region of long bones, with 50–65% of the most often localizing around the knee [3]. Extended curettage has increasingly become the preferred treatment for GCTBs, but the high recurrence rate (20–30%) after curettage poses a major challenge for orthopaedic surgeons [4–6].

Some studies have found pre-operative computed tomography (CT) to be valuable in evaluating bone involvement of GCTBs and predicting their recurrence [7, 8]. Puthoor et al. [9] found that patients having prior CT classification had a significantly lower long-term recurrence rate compared to those without pre-operative CT (i.e., 12.9% CT classified versus 30% non-CT classified). Certain features of GCTBs on CT images have been selected to develop a prediction radiomics model in spinal GCTBs, achieving 89% ability to predict local recurrence [10]. However, while GCTBs predominantly arise around the knee joint, only a few studies have specifically focused on this region using CT [8, 11]. Those CT studies found that patients showed a higher recurrence rate with cortical-bone involvement or with short tumour-articular distances, but these rates did not reach statistical significance [12, 13]. Residual tumor tissue related to GCTBs post-surgery was usually considered responsible for local recurrence in these studies, but they did not consider possible surgical factors as causative [12]. Thus, in the present study, by using appropriate statistical methodologies and taking surgical approach factors into consideration, we adopted a more refined method to analyze pre-operative CT images of primary GCTBs around the knee, and we included other risk factors to predict recurrence.

The aims of this study were (1) to analyze the local recurrence rate for primary GCTBs occurring around the knee treated after extended curettage; (2) to identify and analyze the features of GCTBs around the knee on CT images related to recurrence.

## Methods

**Fig. 1:**
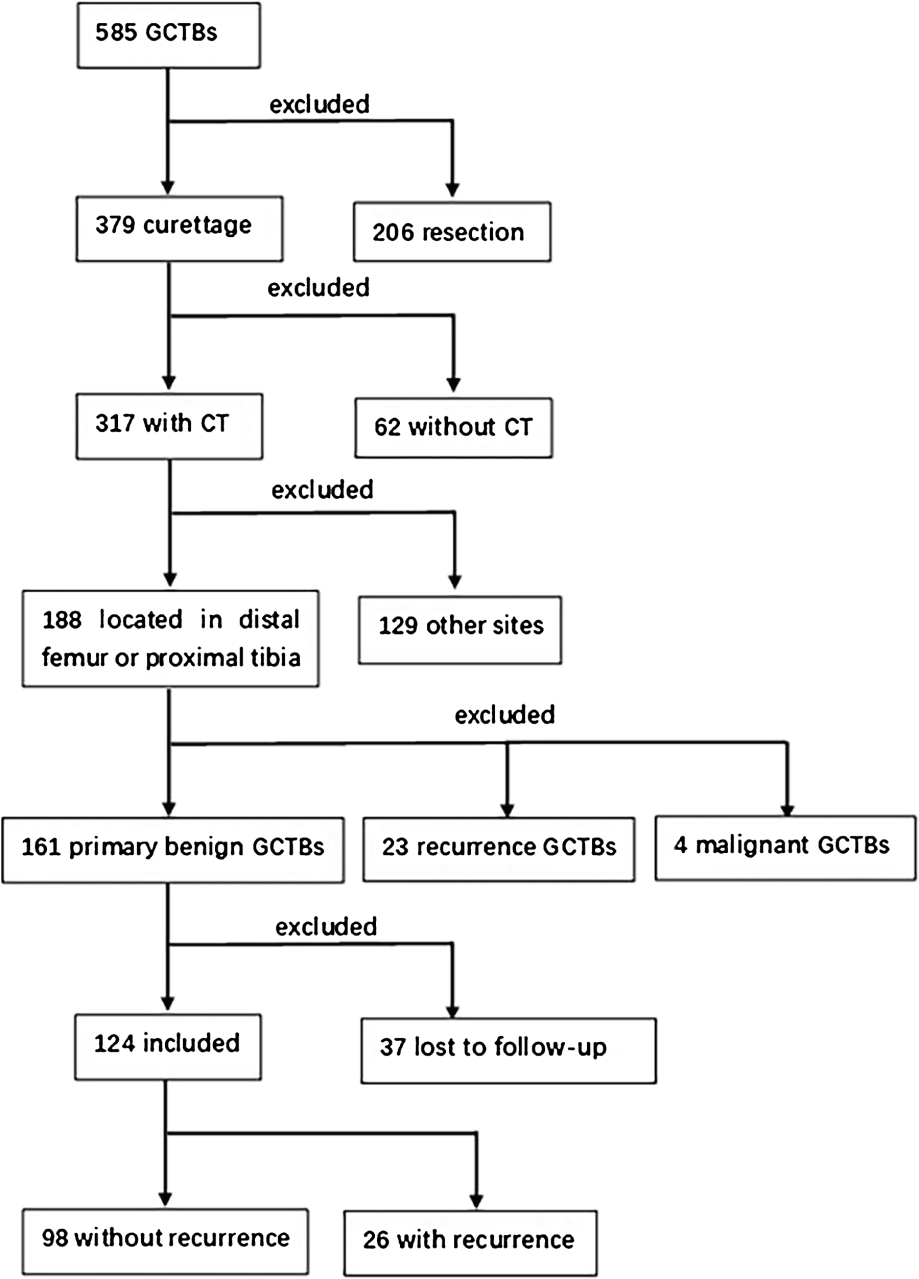
Flow chart of the study

For the period between November 2010 and June 2019, 585 patients diagnosed with giant cell tumour of bone were treated in our institution. We retrospectively evaluated records of all patients (*n* = 188) who underwent extended intralesional curettage for GCTB located in the distal femur or proximal tibia and who had available CT imaging records. A total of 161 patients received a histopathological diagnosis of primary benign GCTB, and 23 patients had a recurrence of GCTB; four patients with malignant GCTB were excluded. Of the 161 patients, 23% (37 patients) were lost to follow-up in the last year; we followed up the remaining patients for at least 24 months. Among the included patients, the average follow-up time was 69.5 months (range: 24–127 months). Finally, a total of 124 patients (64 women, 60 men) were included for analysis; the detailed flow chart of the study is shown in Fig. 1. The average patient age at surgery was 36.4 years (range: 14–72 years). Table 1 summarizes demographic, clinical, and tumour characteristics of the included patients at baseline.

**Table 1:** Demographics and baseline characteristics of included patients with GCTB (*n* = 124)

	Recurrence*					
Variable	Yes	No	Recurrence rate (%)	Odds ratio (95% CI)	Mean difference (95% CI)	*P*-value
Sex, no. (%)						
Male	16 (61.5)	44 (44.9)	26.7	1.96 (0.81–4,76)	/	0.13
Female (reference)	10 (38.5)	54 (55.1)	15.6			
GCTB location, no. (%)						
Distal femur	16 (61.5)	51 (52.0)	23.9	1.48 (0.61–3.57)	/	0.39
Proximal tibia (reference)	10 (38.5)	47 (48.0)	17.5			
Side, No. (%)						
Left	14 (53.8)	47 (48.0)	23.0	1.27 (0.53–3.01)	/	0.59
Right (reference)	12 (46.2)	51 (52.0)	19.0			
Campanacci classification, no. (%)						
Grade I	0 (0.0)	2 (2.0)	0	/	/	
Grade II	13 (50.0)	57 (58.2)	18.6	0.68 (0.29–1.63)	/	0.39
Grade III (reference)	13 (50.0)	39 (39.8)	25.0			
Previous CT classification, no. (%)						
Class 1	9 (34.6)	53 (54.1)	14.5	0.34 (0.05–2.14)	/	0.20
Class 2	15 (57.7)	41 (41.8)	26.8	0.73 (0.12–4.42)	/	
Class 3 (reference)	2 (7.7)	4 (4.1)	33.3			
Pathological fracture, no. (%)						
Yes	5 (19.2)	10 (10.2)	33.3	2.10 (0.65–6.78)	/	0.21
No (reference)	21 (80.8)	88 (89.8)	19.3			
Secondary ABC, no. (%)						
Yes	10 (38.5)	33 (33.7)	23.3	1.23 (0.50–3.01)	/	0.65
No (reference)	16 (61.5)	65 (66.3)	19.8			
Preoperative denosumab, no. (%)						
Yes	3 (11.5)	20 (20.4)	13.0	0.51 (0.14–1.87)	/	0.40
No (reference)	23 (88.5)	78 (79.6)	22.8			
Postoperative denosumab, no. (%)						
Yes	2 (7.7)	18 (18.4)	20.0	0.37 (0.08–1.71)	/	0.24
No (reference)	24 (92.3)	80 (81.6)	23.1			
Postoperative bisphosphonate, no. (%)						
Yes	2 (7.7)	12 (12.2)	14.3	0.60 (0.13–2.85)	/	0.73
No (reference)	24 (92.3)	86 (87.8)	21.8			
Cavity reconstruction, no. (%)						
Cement alone	1 (3.8)	7 (7.1)	12.5	0.43 (0.04–4.64)	/	0.78
Bone graft alone	21 (80.8)	79 (80.6)	21.0	0.80 (0.23–2.73)	/	
Cement + bone graft (reference)	4 (15.4)	12 (12.2)	25.0			
Distance between tumor edge and articular surface, no. (%)						
< 2 mm	20 (76.9)	46 (46.9)	30.3	3.77 (1.39–10.20)	/	0.006
≥ 2 mm (reference)	6 (23.1)	52 (53.1)	10.3			
Destruction of posterior cortical bone, no. (%)						
Yes	14 (53.8)	21 (21.4)	40.0	4.28 (1.72–10.62)	/	0.001
No (reference)	12(46.2)	77 (78.6)	15.6			
Age, year (mean ± SD) †	28.50 ± 9.31	38.50 ± 14.17	/	/	-10.00 (-14.64 to -5.36)	< 0.001
Follow-up, months (mean ± SD)	81.54 ± 30.67	66.31 ± 30.69	/	/	15.23 (1.84 to 28.63)	0.026
Ki-67 proliferative index, % (mean ± SD)	18.27 ± 10.02	15.33 ± 8.18	/	/	2.94 (-0.81 to 6.69)	0.12
Size of tumor, mm (mean ± SD)	57.71 ± 15.50	53.47 ± 14.79	/	/	4.24 (-2.28 to 10.77)	0.20

^*^Minimum follow-up was 24 months; mean ± SD follow-up was 69.5 ± 31.2 months (range: 24–127 months). † Age at surgery for extended curettage

*ABC*, Aneurysmal bone cyst

### Data sources and follow-up

Patient information was obtained from the hospital’s clinical inpatient database system and medical records. Data collected included age, sex, location of the tumour, and treatment details. Histopathological analysis revealed that 34.7% (43/124) of GCTBs were complicated by an aneurysmal bone cyst (ABC). We used the Ki-67 proliferative index to evaluate the growth fraction of GCTB neoplastic cell populations [14]; immunohistochemical data for calculating this index was collected from biopsy tissue (*n* = 82) or from surgical pathology (*n* = 42) when a biopsy was not performed. We followed the procedures outlined in Cheng et al. [15]. Biopsy was routinely performed, and some patients with explicit imaging features of benign tumour and specifically GCTB (typically lytic and eccentric; extending to the articular cartilage; a well-defined, non-sclerotic border and no matrix mineralization [7]) would receive curettage without biopsy, and the surgeon would also send intra-operative frozen sections as a precaution.

Patients received follow-up primarily through clinic visits and telephone calls or through WeChat (a widely available and used instant messaging software). Clinic visits were recommended one, three, six and 12 months for the first year post-surgery, and then annually thereafter. Standard radiographic evaluations were obtained in post-operative examinations, and additional CT or enhanced MRIs were performed when recurrence was suspected. Recurrence was considered when osteolytic destruction around graft bone or PMMA, soft tissue mass formation, and expansile change on imaging. Patients in our series who experienced tumour recurrence had received puncture biopsy or re-operation, and recurrence was confirmed histologically.

### Adjuvant therapy

In the present study, 23 patients were treated pre-operatively with denosumab systemically, and 20 patients were treated post-operatively with denosumab. Fourteen patients received post-operative bisphosphonate therapy. The details (dosage, duration, etc.,) of adjuvant therapy involving denosumab and bisphosphonates were not available for our review in our follow-up.

### Image analysis

**Fig. 2:**
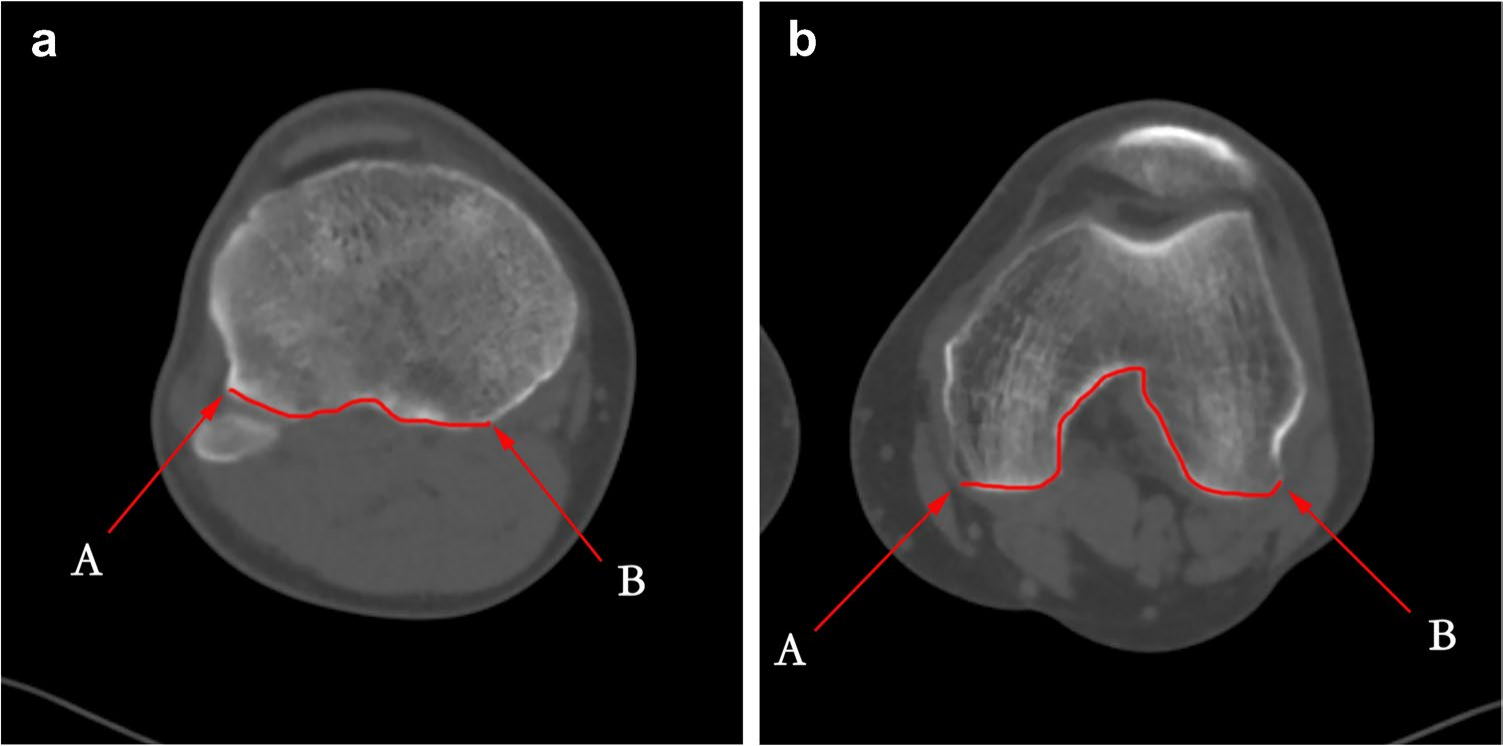
Transverse CT images of the knee joint with superimposed measurement boundaries (red). **a** Proximal tibia and fibular head showing the anterior-most point of the fibular head (arrow A, end of line segment) and the posteromedial ridge of the proximal tibia (arrow B, end of the line segment). The red superimposed line represents the posterior boundary of the posterior cortex of the proximal tibia. **b** Posterolateral (arrow A, end of the line segment) and posteromedial (arrow B, end of the line segment) parts of the femoral condyle. The red superimposed line represents the posterior boundary of the posterior cortex of the distal femur

**Fig. 3:**
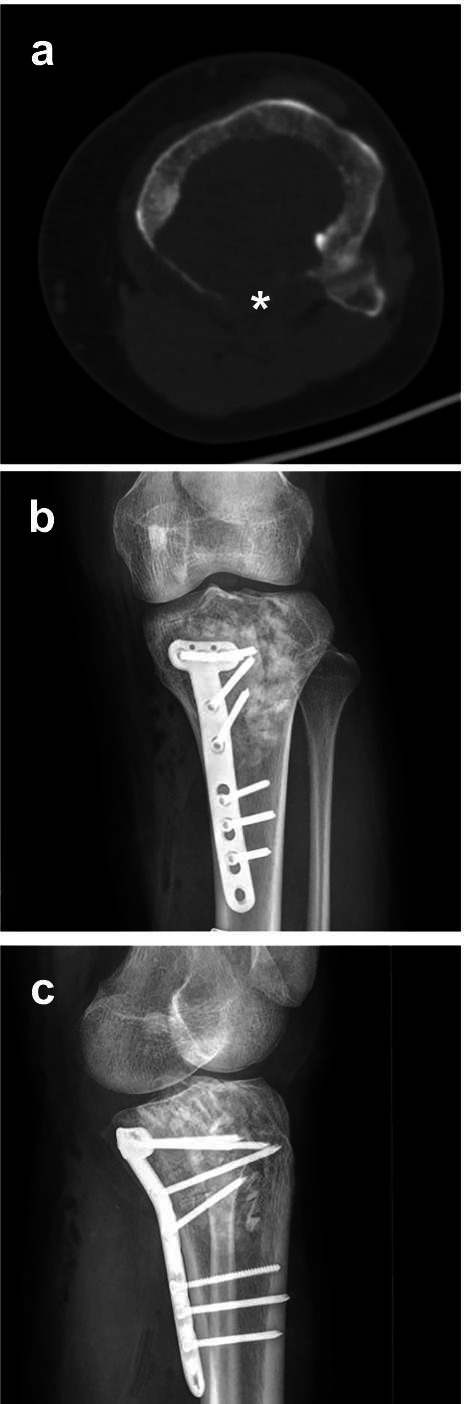
CT and X-ray images of a 26-year-old woman’s knee joint with histopathologically confirmed GCTB. **a** Transverse CT image showing the destruction of the posterior cortex of the proximal tibia (DPC, white asterisk). The patient received extended curettage through a posterior approach. **b**, **c** Anteroposterior and lateral X-ray images, respectively, show no sign of recurrence during follow-up 64 months later. This patient was implanted with a fixation device to stabilize the joint

**Fig. 4:**
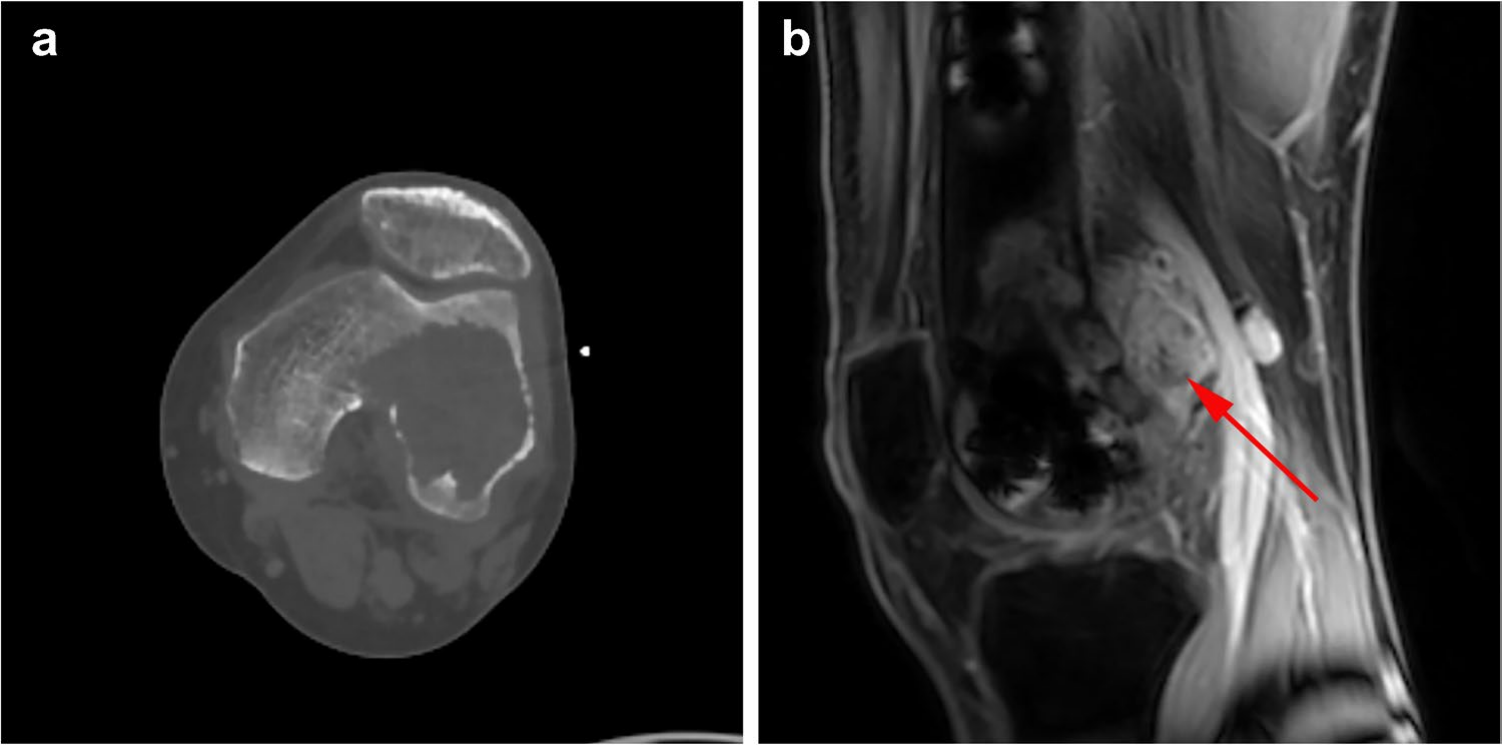
CT and MRI images of left knee joint showing GCTB in a 25-year-old man. The tumor recurred 22 months after follow-up. **a** Transverse CT image showing the destruction of the posterior cortex of the distal femur. The patient received extended curettage through an anterolateral approach. **b** Sagittal enhanced MRI image of the knee joint revealed recurrent soft mass (red arrow) within the popliteal fossa

For this study, we obtained all high-resolution CT images (slice thickness: 0.625 mm) from our hospital's picture archive and communication system (PACS). To identify and analyze features of GCTBs that could be related to recurrence, we quantified several parameters in the CT images. First, we measured the distance between the tumour edge and articular surface (DTA) in CT images using a method similar to that described by Zhou et al. [8]. Briefly, the posterior area of the proximal tibia was categorized using a “three-column concept,” according to Luo et al. [16]. This concept refers to a three-dimensional classification system of a bone anomaly, in this case, the GCTB, that uses lateral, medial, and posterior “columns” in the proximal tibia shown in CT images to more precisely determine the morphology and extent of the tumour. The posterior cortex of the distal femur was defined as the area between the posterolateral and posteromedial parts of the femoral condyles (Fig. 2). Destruction of posterior cortical bone (DPC) was defined as follows: the cortex and rim of reactive bone in the above-mentioned areas were rather thin and moderately expanded (i.e., appearance likened to soap bubbles [17]), or the bone cortex integrity was deficiency with or without extraosseous soft-tissue extension in the above-mentioned areas (Figs. 3a and 4a).

These parameters were identified and analyzed independently in the CT images by three experienced orthopaedic oncologists. For the DTA parameter, the measurements of the three oncologists were averaged, and the average value was taken as the final recorded value. Disagreements among the three oncologists were settled by majority opinions. The orthopaedic oncologist raters were blinded to the patients’ clinical information. GCTBs were graded on radiograph according to the Campanacci et al. classification system [18]. On CT images, GCTBs were classified into three classes according to a previous study: class 1 lesion was intraosseous with no cortical breaks. Class 2 and class 3 lesion was extraosseous with cortical breaks. Class 2 lesion was not exceeding one-third of the bone’s circumference, while class 3 lesion extended into more than one-third of the bone’s circumference or broke through the cortex at more than one surface [9].

### Surgical procedures

Four surgical teams at a single institution performed the surgeries following similar operative procedures. The surgeon of each team decided which surgical approach was suitable, based mainly on pre-operative imaging results and the surgeon’s experience. For extended curettage, the entire tumour cavity was exposed over the affected bone through a large cortical window. Then, we used a set of standard bone curettes to thoroughly remove the visible lesion. The residual bony margin was debrided and extended using high-speed burring. For better visualization, the area was continuously flushed with sterile saline. Next, the cavity was cauterized with an adjuvant (phenol or hydrogen peroxide) in order to destroy any remaining microscopic remnants of the GCTB within the cortex and to minimize the possibility of local recurrence. For bone reconstruction, we filled the cavity with polymethylmethacrylate (PMMA); autologous bone (e.g., autologous iliac bone); allogeneic bone; or a combination of PMMA and bone graft. For cases reconstructed with the PMMA-bone graft combination, we implanted the bone graft in the subchondral region, and then we filled the cavity with cement. Of the included patients, eight received PMMA only, 100 received a bone graft (autologous or allogeneic), and 16 received PMMA plus a bone graft.

### Statistical analysis

Statistical analysis was performed using SPSS software for Windows, version 26 (IBM, Armonk, NY, USA). All *p*-values were two-sided, and a *p*-value of < 0.05 was taken as significant. The precision of quantitative data values was rounded to the nearest tenth or hundredth places; data are presented as means ± standard deviations (SD) unless otherwise indicated. We used Student’s *t*-test to analyze continuous variables, and Pearson’s chi-square test or Fisher’s exact test to compare differences between categorical variables. Subgroup analyses were performed on the data of patients who had destruction of the posterior cortical bone. We used the AIC to identify which variables were to be entered into multivariate logistic regression models to determine significant factors affecting local recurrence [19]. Kaplan–Meier curves were made to evaluate the recurrence risk.

## Results

The overall local recurrence rate after extended curettage was 21.0% (26/124), with an average surgery-recurrence interval of 20.7 ± 13.9 months (range, 3–63 months). Local recurrence-free survival at one year was 5.6% and at 2 years was 16.1%. The average age in the recurrence group was significantly younger compared to that in the non-recurrence group (28.50 versus 38.50 years, *p* < 0.001). The average Ki-67 proliferation index (recurrence: 18.27%; non-recurrence, 15.32%, *p* = 0.12) and the average tumour size (recurrence: 57.72 mm; non-recurrence: 53.47 mm, *p* = 0.20) of the two groups were not significantly different. GCTBs with DTAs < 2 mm were significantly related to recurrence (Pearson’s chi-square, *p* = 0.006). Similarly, DPC was significantly related to recurrence (Pearson’s chi-square, *p* = 0.001) (Table 1). No other demographic or clinical characteristics of patients were significantly related to recurrence. Recurrence was seen in 12.5% (1 patient) of cement only group, 21.0% (21 patients) of bone graft group, and 25.0% (4 patients) of cement + bone graft group. In the group with adjuvant treatment, the recurrence rate was 13.0% (three of twenty-three) in patients who received pre-operative denosumab, 10.0% (two of twenty) in those who received post-operative denosumab, and 14.3% (two of fourteen) in those who received post-operative bisphosphonate (Table 1). In the subgroup analyses, the surgical approach used for extended curettage had a significant risk difference (RD) for tumour recurrence (posterior approach: RD =  − 50% [95% CI: − 31.48 to − 68.52%]) (see Supplementary Table S1).

**Fig. 5:**
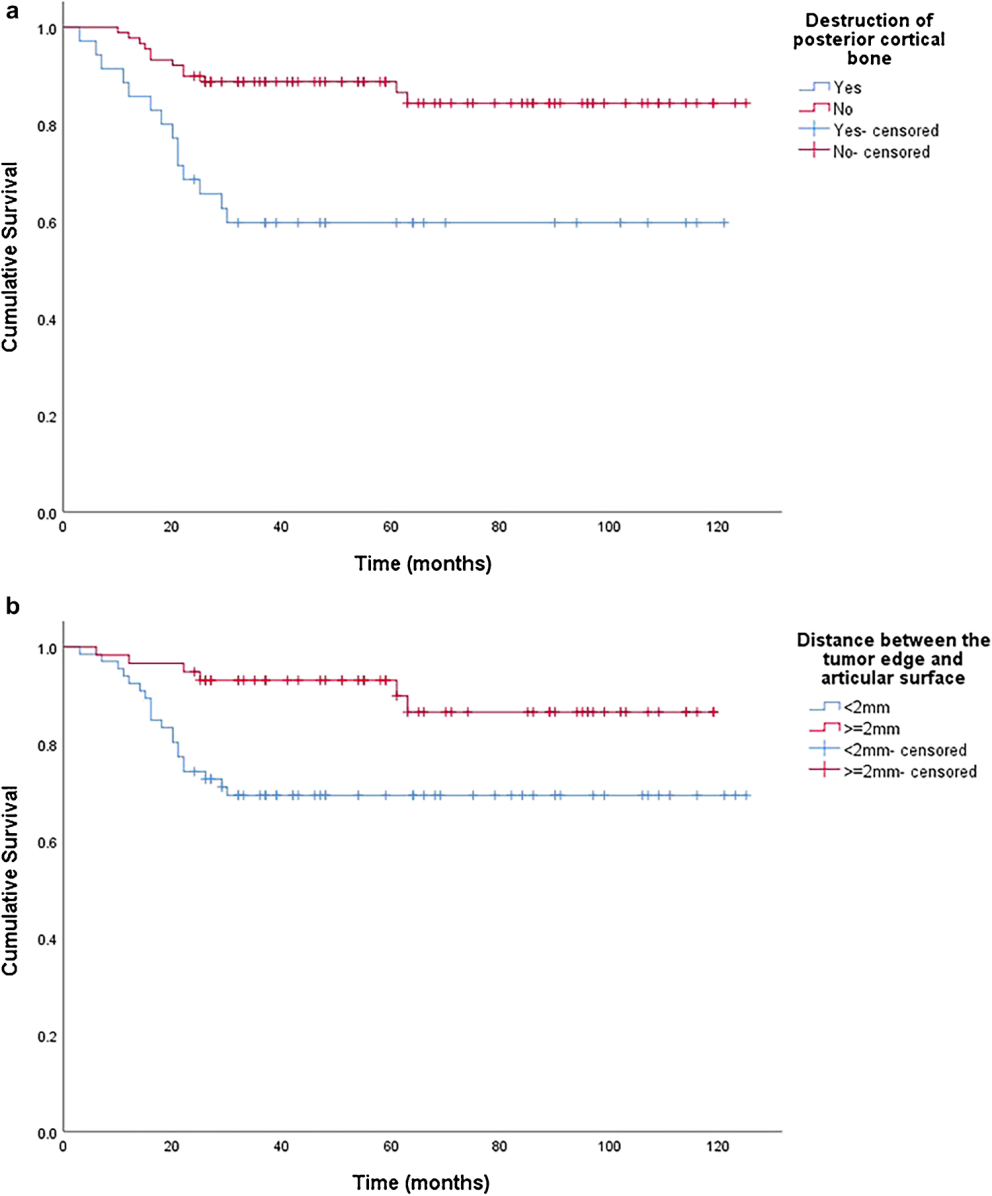
Kaplan–Meier survival curves. **a** Worse prognosis for recurrence-free survival in patients when the posterior cortical bone was destructed (HR, 3.50 (1.62 7.57); *p* = 0.001). **b** Worse prognosis for recurrence-free survival in patients when the distance between tumour edge and the articular surface was less than 2 cm (HR, 3.36 (1.35 8.37); *p* = 0.006)

To determine which possible risk factors were associated with local recurrence, we performed multivariate logistic regression analysis based on the minimal AIC. The risk factors associated with GCTB recurrence were patient age at surgery, DTA, and DPC (AIC = 95.01). Age was entered as a covariate in the logistics regression analysis. Table 2 shows that DTA (< 2 mm) and DPC were independent risk factors for GCTB recurrence after extended curettage (*p* < 0.05). The C-index of the final model was 0.79 (95% CI: 0.71 to 0.88). Values greater than 0.7 indicate a good model fit, and indices greater than 0.8 are considered strong models [20]. The Kaplan–Meier survival curves (Fig. 5a and 5b) showed a worse prognosis for recurrence-free survival in patients when the posterior cortical bone was destructed (HR, 3.50 (1.62 7.57); *p* = 0.001) and the distance between tumour edge and the articular surface was less than 2 cm (HR, 3.36 (1.35 8.37); *p* = 0.006). In sensitivity analyses (excluding patients receiving denosumab or bisphosphonate), there showed no clear difference in trends (see Supplementary Tables S2 and S3).

**Table 2:** Results of multivariate logistic regression analyses of factors predicting recurrence

Variable	Odds ratio (95% CI)	*P*-value
Age (per year)	0.94 (0.90–0.98)	0.008
Distance between the tumor edge and articular surface < 2 mm vs. ≥ 2 mm	3.33 (1.14–9.72)	0.028
Destruction of posterior cortical bone Yes vs. no	2.73 (1.01–7.35)	0.047

## Discussion

GCTBs are intermediate bone tumours that have a tendency to recur locally despite surgical removal [18]. It is believed that residual tumour tissue left after surgery is responsible for local recurrence [21]. We hypothesized that, in GCTBs, destruction of posterior cortical bone and invasion of the articular surface of the knee are two factors that may be associated with a higher risk of missing residual tumour tissue following extended curettage via a typical surgical approach. In the present study, multivariate logistic regression analyses identified DTA (< 2 mm) and DPC, in addition to patient age, as factors significantly related to recurrence. Thus, to reduce local recurrence, our results suggest that accurately determining DTA and DPC in GCTBs prior to extended curettage may represent an important step. In this regard, CT imaging is superior to traditional two-dimensional radiographs for assessing bone invasion and destruction of posterior cortical and subchondral bone; it is more difficult to clearly identify this kind of invasion and destruction in traditional 2-D radiographs because structures overlap on the radiographs [22].

The main limitation of our study was that it is retrospectively conducted at a single center. However, to reduce subject-selection bias, we did include all consecutive patients treated at our hospital who met the study criteria. As a national leading orthopaedic institution, we accept a large number of referral patients from across the country, making our study subject to some degree of referral bias. Also, as adjuvant therapy (denosumab, bisphosphonates) has been extensively used for the treatment of GCTBs and denosumab may increase the rate of local recurrence after curettage [4, 23], the details (dosages and frequencies) of adjuvant treatments were not available in our study. Because denosumab was not approved in Mainland China during the study period, all patients had to obtain denosumab in other countries and regions. It was occasionally not available, and some patients were unable to receive denosumab therapy according to guideline recommendations. Thus, we performed sensitivity analyses by excluding each group of patients receiving adjuvant treatments and the results were stable. Lastly, although GCTBs generally recur within two years [24], six cases had recurrence after two years (23.1% of all recurrent patients). Thus, the possibility of recurrence might still exist for patients who lacked follow-up beyond 24 months.

In the current study, we found the local recurrence rate was consistent with previous studies (Table 3) after extended curettage combined with adjuvant therapy [13, 25–29]. Although with a lower risk of tumour recurrence, en bloc resection has a greater functional impairment and higher incidence of complications [30]. Joint-preserving intralesional curettage has increasingly become the first choice to retain maximum limb function and achieve a high quality of life, especially common in young patients [31]. As with other authors, the univariate analysis results showed that the recurrence rate did not correlate with sex, pathological fracture, or Campanacci classification [4, 32, 33]. Multiple early studies in the 1990s revealed that cementation might reduce the risk of GCTB recurrence [34, 35]. However, according to recent proceedings, PMMA was no longer considered to have a locally anti-tumour effect and, instead, only a method of mechanical enforcement of the tumoral cavity [36]. Our results also showed no significant difference between the groups of cavity reconstruction. Puthoor et al. [9] classified GCTB into three classes based on CT findings, and they only used it for surgical determination but not for prognostic analysis. In our study, a large absolute but statistically insignificant difference was found in local recurrence rate among groups according to the previous CT classification (class 1, 14.5% vs. class 2, 26.8% vs. class 3, 33.3%), and its clinical significance needs further investigation with more large-scale research. Young people have a higher risk of recurrence, and our study is coincides with the report by Klenke et al. [21] and Kivioja et al. [37].

**Table 3:** The relevant series reporting on local recurrence rate of giant cell tumor of knee with extended curettage

Study (year)	Patients, (*n*)	Local recurrence rate	Follow-up (years) (range)	Number of institutions
Jamshidi et al. [26] (2021)	20	20%	5.5 (2–22)	Single center
Hu et al. [27] (2016)	181	36%	4.9 (2–16)	Multicenter
Kafchitsas et al. [28] (2010)	38	37%	8.7 (2–16)	Single center
Teng et al. [29] (2019)	104	11%	2.8 (1–8)	Single center
Prosser et al. [13] (2005)	104	19%	5.8 (2–18)	Single center
He et al. [25] (2018)	55	42%	/(2–12)	Single center
This study	124	21%	5.8 (2–11)	Single center

Other clinical factors may also increase the risk of recurrence. McGough et al. [38] observed that subchondral GCTBs commonly recur, and Suzuki et al. [39] identified an inverse relationship between subchondral bone thickness and recurrence on the articular-surface side. These conclusions were confirmed by our present finding that DTA is a risk factor for recurrence in patients with GCTB of the knee joint. GCTBs occur mostly in the epi-metaphysis of long bones and usually extend to subchondral bone [29]. When subchondral bone is extensively invaded by the tumour, balancing complete tumor removal and preserving articular cartilage can present a surgical dilemma [29]. In such cases, surgeons have limited options, and thus can scrape away only a small amount of affected bone on the joint side [8]. Thus, it is imperative to accurately evaluate the condition of subchondral bone, as failure to do so might lead to insufficient removal of a lesion [38].

GCTBs usually appear as eccentrically developed lesions [7]. Thus, surgeons generally use a medial or lateral approach depending on the side more affected by the tumour, and rarely use a posterior approach, unless the tumour only invades posterior cortical bone [9]. Critical structures, such as nerves and blood vessels, can adhere to adjacent tumour-affected cortical bone, it can be especially difficult for surgeons to scrape and grind the posterior cortical bone and completely remove soft tumour tissue extending to the posterior side. According to Puthoor et al. [9], removal of GCTBs is better approached for curettage through the site of the cortical break, based on CT. When GCTBs invade only the posterior cortical bone, a posterior approach might be advantageous, as it allows sufficient exposure of the operative field. In addition, through a posterior approach, surgeons could avoid deleterious neurovascular complications caused by non-contact thermal necrosis of PMMA [40]. In our series, seven patients received extended curettage through a posterior approach (2 femora, 5 tibias), and none of them had a local recurrence with a minimum follow-up of 24 months. Given the small number of patients, further large-scale prospective studies would be needed to draw a firm conclusion about this issue.

## Conclusion

To our knowledge, this is the first study of demographic, clinical, and CT-determined risk factors associated with recurrence in patients with primary GCTB around the knee. We found a higher local recurrence rate in younger patients, greater destruction of posterior cortical bone, and a shorter distance between the tumor edge and articular surface on CT. For the contemporary treatment of GCTB, it is vital to stratify patients by the risk of recurrence. High-risk patients require more careful pre-operative assessment and more rigorous post-operative monitoring.

## Supplementary Information

Below is the link to the electronic supplementary material.

Supplementary file1 (DOCX 21 kb)

## Data Availability

Not applicable.
