# Supplementary material for: Computerised tomography features of giant cell tumour of the knee are associated with local recurrence after extended curettage
Source: Int Orthop. 2021 Nov 16;46(2):381–90. doi: 10.1007/s00264-021-05260-6 (PMC8782792; doi:10.1007/s00264-021-05260-6)
Supplement: Supplementary file 1 — Supplementary file1 (DOCX 21 kb) [file 264_2021_5260_MOESM1_ESM.docx]

**Table S1**. Subgroup analysis on patients with destruction of posterior cortical bone (n = 35).

|  | Recurrence | |  |  |  |  |
| --- | --- | --- | --- | --- | --- | --- |
| Variable | Yes | No | Risk difference of recurrence (95% CI) | Odds ratio (95% CI) | Mean difference (95% CI) | P value |
| Posterior approach, No. (%) |  |  |  |  |  |  |
| Yes | 0 (0.0) | 7 (33.3) | -50% (-31.48% – -68.52%) | / | / | / |
| No (reference) | 14 (100.0) | 14 (66.7) |  |  |  |  |
| DTA*, No. (%) |  |  |  |  |  |  |
| <2 mm | 12 (85.7) | 12 (57.1) | / | 4.5 (0.80–25.35) | / | 0.14 |
| ≥2 mm (reference) | 2 (14.3) | 9 (42.9) |  |  |  |  |
| Age, yr (mean ± SD) | 27.86 ± 9.31 | 34.29 ±14.80 | / | / | -6.43 (-14.72 – 1.87) | 0.12 |

* DTA, distance between the tumor edge and articular surface.

**Table S2.** Sensitivity analysis of univariate analysis after excluding patients receiving denosumab treatment, n=88)

|  | Recurrence | | | |  | |  | |  | |  |
| --- | --- | --- | --- | --- | --- | --- | --- | --- | --- | --- | --- |
| Variable | Yes | | No | | Odds ratio (95% CI) | | Mean difference (95% CI) | | P value | |  |
|  |  | |  | |  | |  | |  | |  |
| Distance between tumor edge and articular surface, No. (%) | |  | |  | |  | |  | |  | |
| <2 mm | | 16 (72.7) | | 31 (47.0) | | 3.01 (1.05–8.65) | | / | | 0.036 | |
| ≥2 mm (reference) | | 6 (27.3) | | 35 (53.0) | |  | |  | |  | |
| Destruction of posterior cortical bone, No. (%) | |  | |  | |  | |  | |  | |
| Yes | | 12 (54.5) | | 13 (19.7) | | 4.89 (1.74–13.78) | | / | | 0.002 | |
| No (reference) | | 10 (45.5) | | 53 (80.3) | |  | |  | |  | |
|  |  | |  | |  | |  | |  | |  |
| Age, yr (mean ± SD) | | 26.64 ± 7.76 | | 38.89 ± 14.72 | | / | | -12.26 (-17.15 – -7.36) | | <0.001 | |

**Table S3.** Sensitivity analysis of univariate analysis after excluding patients receiving bisphosphonate treatment, n=110)

|  | Recurrence | | | |  | |  | |  | |  |
| --- | --- | --- | --- | --- | --- | --- | --- | --- | --- | --- | --- |
| Variable | Yes | | No | | Odds ratio (95% CI) | | Mean difference (95% CI) | | P value | |  |
|  |  | |  | |  | |  | |  | |  |
| Distance between tumor edge and articular surface, No. (%) | |  | |  | |  | |  | |  | |
| <2 mm | | 18 (75.0) | | 40 (46.5) | | 3.45 (1.25–9.53) | | / | | 0.013 | |
| ≥2 mm (reference) | | 6 (25.0) | | 46 (53.5) | |  | |  | |  | |
| Destruction of posterior cortical bone, No. (%) | |  | |  | |  | |  | |  | |
| Yes | | 12 (50.0) | | 18 (20.9) | | 3.78 (1.46–9.81) | | / | | 0.005 | |
| No (reference) | | 12 (50.0) | | 68 (79.1) | |  | |  | |  | |
|  |  | |  | |  | |  | |  | |  |
| Age, yr (mean ± SD) | | 28.04 ± 9.55 | | 39.00 ± 14.47 | | / | | -10.96 (-15.96 – -5.96) | | <0.001 | |
